# Cholestatic Jaundice With the Use of Methylstenbolone and Dymethazine, Designer Steroids Found in Super DMZ Rx 2.0 “Nutritional Supplement”: A Case Report

**DOI:** 10.1177/2324709614532800

**Published:** 2014-04-22

**Authors:** Priscilla Agbenyefia, Christina A. Arnold, Robert Kirkpatrick

**Affiliations:** 1 Ohio State University, Columbus, OH, USA

**Keywords:** methylstenbolone, dymethazine, anabolic androgenic steroids, cholestatic jaundice, DILI

## Abstract

“Nutritional supplements” that promise an increase in muscle mass and strength are becoming a go to item as enhancing one’s physical appearance becomes a more important part of our society. This is alarming because many of these nutritional supplements rely on androgen precursors to deliver their promises, without adequately informing consumers of the potential side effects of such agents. These products may conceal the presence of potent androgens to avoid regulatory sanctions and become more appealing to consumers. Recent reports have shown that some products marketed as “nutritional supplements” have been found to contain androgenic anabolic steroids. Methylstenbolone and dymethazine are new androgenic anabolic steroids currently gaining popularity among body builders for their performance-enhancing properties and rapid effects on muscle mass. These agents are found together in Super DMZ Rx 2.0, a “dietary supplement” for bodybuilders. Here we report the first case of Super DMZ Rx 2.0–induced cholestatic jaundice in a 26-year-old previously healthy Caucasian male, who took the supplement according to the manufacturer’s instructions for 30 days.

## Introduction

A significant percentage of athletes and teenagers use androgenic anabolic steroids (AAS) in their everyday lives. In a study of 718 athletes from 92 Amsterdam fitness centers, 8.2% of the athletes reported using performance-enhancing drugs.^1^ A study of American teenagers found similar results, with 6.6% of 3403 12th-grade students admitting to consumption of AAS.^2,3^ In addition, many over-the-counter dietary supplements that do not list AAS as ingredients may contain AAS, putting consumers at risk for potential adverse effects.^4^ An international study conducted between 2000 and 2001 on 634 nutritional supplements purchased in 13 different countries revealed that 15% of putative nutritional supplements contain AAS.^5^

2,17α-Dimethyl-5α-androsta-1-en-17β-ol-3-one (methylstenbolone) is an AAS used by many “bodybuilding supplement” manufacturers. It has been compared to Superdrol (methyldrostanolone), a competitor that was recently banned by the Food and Drug Administration. Methylstenbolone is currently not a controlled substance in the United States. It is very potent and is prized among its users for giving them a hard, dense, and dry muscular appearance.^6^ 17β-Hydroxy 2α,17β-dimethyl 5α-androstan 3-one azine (dymethazine), also currently not a controlled substance in the United States, is used in body building supplements by manufacturers such as iForce Nutrition. Dymethazine is a conjugate of 2 methyldrostanolone residues and is very popular for its alleged myotropic effects.^6^ Drostanolone, stenbolone, and “all related compounds” are banned substances on the World Anti-Doping Agency list and thus are banned for sports competition.^7^

Super DMZ Rx 2.0 (Blackstone Labs), the “Hardening Stack,” combines 10 mg of methylstenbolone and 10 mg of dymethazine into a capsule to be taken orally.^6^ Consumers of this “dietary supplement” are advised by the manufacturer to take 1 to 2 capsules daily, preferably with food, with 2 capsules being the maximum suggested number of capsules per day. It is also recommended to use Super DMZ Rx 2.0 in cycles, not exceeding 4 weeks of continuous use and with at least 8 weeks in between cycles.^6^

The purpose of this article is to report a case of Super DMZ Rx 2.0–induced cholestatic jaundice in an attempt to emphasize the toxic effects of methylstenbolone and dymethazine on the biliary system and hepatocytes.

## Case Report

A 26-year-old previously healthy Caucasian male presented to our liver service via the emergency department complaining of a 2-week history of mild indigestion, dark brown urine, and clay-colored stools. He had also experienced jaundice for 1 week and pruritus for a few days. Five weeks prior to presentation, the patient had begun taking Super DMZ Rx 2.0. He followed the manufacturer’s instructions, taking 1 tablet twice daily for 30 days. The patient denied inappropriate dosing of the product or use of any recreational or prescription drugs while taking the supplement. At presentation, his last reported Super DMZ Rx 2.0 consumption was 1 week prior, and his last alcohol intake was approximately 45 days prior.

The patient noticed symptoms of dark urine and clay stool 3 weeks after he began taking the product. He later developed jaundice by the end of the 4th week of use, whereupon he discontinued the product, presenting to a local emergency department (ED) on the 5th week when his symptoms failed to resolve. He was discharged from the ED but returned 4 days later complaining of worsening jaundice and pruritus, at which point he was transferred to Ohio State University Wexner Medical Center (OSUWMC). Outside hospital labs showed alanine transaminase (ALT) of 269, aspartate transaminase (AST) of 154, total bilirubin of 19.3, direct bilirubin of 16.4, alkaline phosphatase (ALP) of 132, and an international normalized ratio (INR) of 1.0. At OSUWMC, he had an initial ALT of 167, AST of 81, total bilirubin of 21.8, direct bilirubin of 13, ALP of 112, and an INR of 1.0. Patient’s social history included prior alcohol use varying from 0 to 4 times weekly with 4 to 10 drinks daily, and tobacco use occasionally. He had no family history of liver disease.

Vital signs were stable on presentation. The patient had moderate scleral icterus and jaundice, but had normal heart, lung, abdominal, and neurological exam findings. No stigmata of liver failure were appreciated at presentation.

Chemistries and complete blood count were within normal limits. Urine toxicology screen and acute viral hepatitis panel were negative, and right upper quadrant ultrasound and magnetic resonance cholangiopancreatography were negative for acute pathology. The patient was admitted for a day and discharged on diphenhydramine with instructions for outpatient follow-up. At first discharge, his total bilirubin had decreased to 21.2, with a direct bilirubin of 12.2, ALT of 141, AST of 68, ALP of 116, and INR of 1.0.

**Figure 1.**
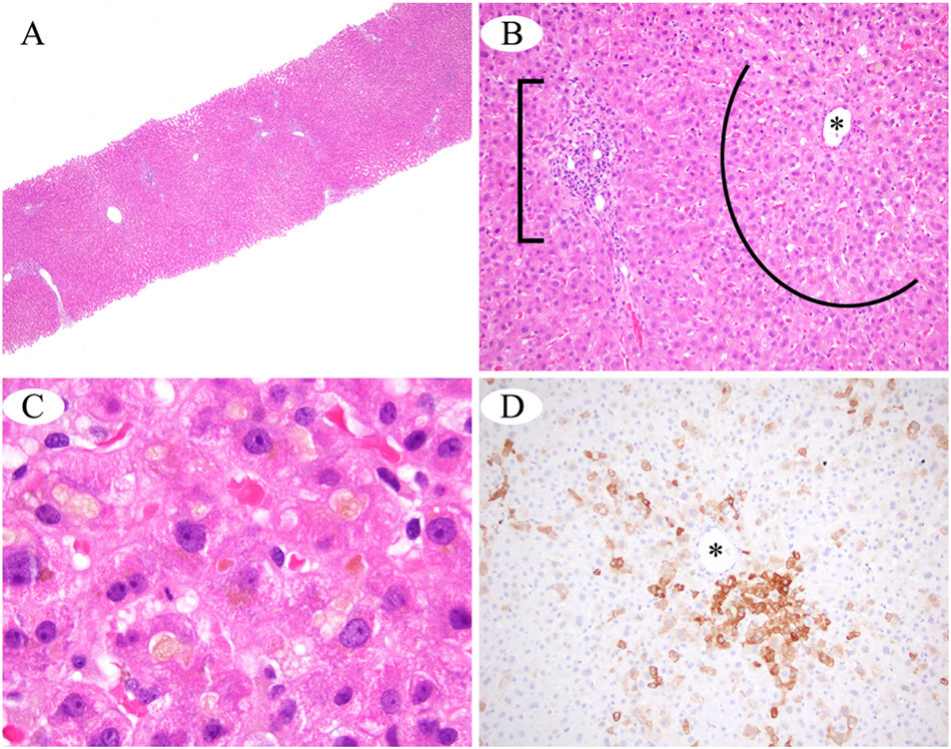
(A) At scanning magnification, essentially normal hepatic architecture is seen; there is no significant steatosis, inflammation, or fibrosis. (B) Cholestasis and mild hepatocellular edema impart a zone of pale discoloration (arc) around the central vein (asterisk), in contrast to the normal eosinophilia of the hepatocytes surrounding the portal tract (bracket). The portal tract shows minimal mixed chronic inflammation. (C) Under oil immersion, the pale yellow bile pigment is seen within hepatocyte cytoplasm and also as discreet canalicular bile plugs. (D) A CK7 immunohistochemical stain highlights the cholestatic hepatocytes surrounding the central vein with brown cytoplasmic immunoreactivity (asterisk).

The patient was readmitted at OSUWMC on day 59 after taking Super DMZ Rx 2.0, for a scheduled liver biopsy, after routine outpatient lab work for acute liver injury 4 days prior showed total bilirubin of 32, direct bilirubin of 28.9, ALT of 121, AST of 101, ALP of 218, and an INR of 1.05. On this admission, he had excessive itching along with unresolved scleral icterus and jaundice. Labs were significant for total bilirubin of 45.9, direct bilirubin of 25.8, ALT of 77, AST of 62, and ALP of 207. INR was 0.9 with a PT of 12.1. The liver biopsy showed canalicular and hepatocellular cholestasis with increase in pigmented macrophages. There were scattered dead hepatocytes and patchy chronic inflammation around the portal vein with lymphocytes, histiocytes, rare eosinophils, and neutrophils (see Figure 1). No significant inflammation, steatosis, or fibrosis was present. Bilirubin decreased to 38.9 a day later and patient was discharged under stable conditions. His recent total bilirubin is 2.1, approximately 10 weeks after he stopped taking Super DMZ Rx 2.0.

## Discussion

Four distinct forms of liver injury have been associated with AAS, ranging from transient increases in serum enzymes to prolonged cholestasis, chronic damage to liver vessels that result in peliosis hepatitis, and hepatic tumors.^8^ Consequently, AAS use can be detrimental to one’s health. This case shows methylstenbolone and dymethazine, AAS found in Super DMZ Rx 2.0, induced cholestasis in a healthy patient who took 1 capsule of the “supplement” twice daily for 30 days as instructed by the manufacturer.

Rise in liver enzymes from AAS induced cholestasis is usually mild as in this case (see Table 1), with elevations in ALT and ALP levels less than 2 to 3 times the upper limits of normal, or even within normal limits despite the presence of deep jaundice.^8^ Typically, AAS-induced cholestasis has an insidious onset, as it takes around 1 to 4 months for injury to the liver to occur after initiating consumption, but can also be delayed for 6 to 24 months.^8^ Presenting symptoms include nausea, fatigue, and pruritus, followed by dark urine and jaundice.^8^ Liver biopsy normally shows bland cholestasis with minimal inflammation and hepatocellular necrosis,^8^ similar to our findings on liver biopsy (see Figure 1). Bland cholestasis is so characteristic of AAS that the diagnosis can be suspected in a patient taking “supplements” to increase muscle mass and strength even if AAS is not disclosed on the label.^8^

**Table 1.** Results of Liver Function Tests, Bilirubin, and INR From Day 35 After First Day of Consuming Super DMZ Rx 2.0 to Day 110.

Day	AST (10-34 IU/L)	ALT (10-40 IU/L)	ALP (44-147 units/L)	Total Bilirubin (0.3-1.9 mg/dL)	Direct Bilirubin (0-0.3 mg/dL)	INR (0.8-1.2)
Day 35	154	269	132	19.3	16.4	1.0
Day 42	81	167	112	21.8	13.0	1.0
Day 43	68	141	116	21.2	12.2	1.0
Day 55	101	121	218	32.0	28.9	1.1
Day 59	62	77	207	45.9	25.8	0.9
Day 60	70	82	201	38.7	23.1	0.9
Day 89	138	174	268	17.6	15.6	1.0
Day 110	75	119	146	2.1	1.4	1.1

Abbreviations: AST, aspartate transaminase; ALT, alanine transaminase; ALP, alkaline phosphatase; INR, international normalized ratio.

The patient’s early symptom of dark urine and clay-colored stools followed by pruritus and jaundice about 3 to 4 weeks after he began taking Super DMZ Rx 2.0, in addition to results from liver function tests and liver biopsy, supports a diagnosis of AAS-induced cholestasis. Other causes of acute liver injury such as viral, ischemic, autoimmune, obstructive, and toxic etiologies were evaluated and excluded in this case. While no re-challenge with Super DMZ Rx 2.0 was recommended or carried out to establish it as the culprit, that was the only medication the patient was taking, and discontinued use lead to a gradual decrease in liver enzymes.

## Conclusion

The use of AAS may continue to rise as more people strive to increase their muscle mass for competition or aesthetic purposes. Supplements like Super DMZ Rx 2.0 are relatively inexpensive, widely available online and over-the-counter, and unregulated. This is problematic because consumers are at risk of severe liver injury due to AAS, even when taken at manufacturer-recommended dosages. Although the manufacturer of Super DMZ Rx 2.0 did not conceal methylstenbolone and dymethazine as the major ingredients of the product, unregulated AAS use is unsafe. It is important for physicians to be aware of this while considering causes of jaundice since AAS-induced liver injury can become fulminant. This report represents the first known case of cholestatic jaundice in a patient taking Super DMZ Rx 2.0 “nutritional supplement.”
